# Differential Enhancement of Fat-Soluble Vitamin Absorption and Bioefficacy via Micellization in Combination with Selected Plant Extracts In Vitro

**DOI:** 10.3390/nu17020359

**Published:** 2025-01-20

**Authors:** Stefanie Steinbauer, Melanie Wallner, Lisa-Marie Karl, Theresa Gramatte, Katja Essl, Marcus Iken, Julian Weghuber, Bernhard Blank-Landeshammer, Clemens Röhrl

**Affiliations:** 1 FFoQSI GmbH—Austrian Competence Centre for Feed and Food Quality, Safety and Innovation, Technopark 1D, 3430 Tulln, Austria; stefanie.steinbauer@fh-wels.at (S.S.); melanie.wallner@fh-wels.at (M.W.); theresa.gramatte@fh-wels.at (T.G.); julian.weghuber@fh-wels.at (J.W.); bernhard.blank-landeshammer@fh-wels.at (B.B.-L.); 2 Center of Excellence Food Technology and Nutrition, University of Applied Sciences Upper Austria, Stelzhamerstraße 23, 4600 Wels, Austria; lisa_karl@t-online.de (L.-M.K.); k.essl@icloud.com (K.E.); 3 PM International AG, 15 Waistrooss, 5445 Schengen, Luxembourg; marcus.iken@pm-international.com

**Keywords:** bioavailability, intestinal bioefficacy, buccal absorption, intestinal absorption, micellization, vitamin–plant–compound interactions, cholecalciferol, ergocalciferol, alpha tocopherol acetate, menaquinone-7

## Abstract

**Background/Objectives:** Individuals with special metabolic demands are at risk of deficiencies in fat-soluble vitamins, which can be counteracted via supplementation. Here, we tested the ability of micellization alone or in combination with selected natural plant extracts to increase the intestinal absorption and bioefficacy of fat-soluble vitamins. **Methods:** Micellated and nonmicellated vitamins D3 (cholecalciferol), D2 (ergocalciferol), E (alpha tocopheryl acetate), and K2 (menaquionone-7) were tested in intestinal Caco-2 or buccal TR146 cells in combination with curcuma (*Curcuma longa*), black pepper (*Piper nigrum*), or ginger (*Zingiber officinale Roscoe*) plant extracts. The vitamin uptake was quantified via HPLC-MS, and bioefficacy was assessed via gene expression analyses or the Griess assay for nitric oxide generation. **Results:** Micellization increased the uptake of vitamin D into buccal and intestinal cells, with vitamin D3 being more efficient than vitamin D2 in increasing the expression of genes involved in calcium transport. The micellization of vitamin E acetate increased its uptake and conversion into biologically active free vitamin E in intestinal cells only. The vitamin K2 uptake into buccal and intestinal cells was increased via micellization. Plant extracts increased the uptake of select micellated vitamins, with no plant extract being effective in combination with all vitamins. The curcuma extract increased the uptake of vitamins D2/D3 but not their bioefficacy. Black pepper and ginger extracts increased the uptake of vitamin E acetate into intestinal cells but failed to increase its conversion into free vitamin E. The ginger extract augmented the uptake of vitamin K2 and increased NO generation additively. **Conclusions:** Our data substantiate the positive effects of micellization on fat-soluble vitamin absorption and bioefficacy in vitro. While the application of plant extracts in addition to micellization to further increase bioefficacy is an interesting approach, further studies are warranted to understand vitamin-specific interactions and translation into increased bioefficacy.

## 1. Introduction

Fat-soluble vitamins D, E, and K are crucial for numerous biological functions, including bone, neuronal, cardiovascular, and intestinal health [1,2,3]. However, the hydrophobic nature of fat-soluble vitamins can challenge their bioavailability, i.e., only a fraction of the ingested vitamins become systemically available and biologically active. The bioavailability of fat-soluble vitamins is characterized by high intra- and interindividual variabilities [4,5,6]. Individuals with special demands, such as elderly individuals, growing children, and pregnant or breastfeeding women, are at risk of deficiencies in fat-soluble vitamins [7,8], making supplementation inevitable.

Both nanoformulations and phytochemicals, termed bioenhancers, have been shown to increase the solubility and bioavailability of numerous health-promoting bioactive reagents [9]. Lipid-based nanocarriers such as liposomes and micelles have been extensively studied because of their low toxicity [10] and superior bioavailability to that of conventional vitamin formulations in clinical trials [11,12]. Curcuma extract, a well-described bioactive mixture of phytochemicals with several health-beneficial properties [13], was shown to increase the cellular uptake of the carotenoids, lutein and zeaxanthin, in Caco-2 cells [14]. Piperine, the most abundant and most active compound in black pepper, is known for its bioavailability-enhancing effects on various drugs and bioactive compounds [15]. Likewise, ginger is known to increase bioavailability [16]. However, many aspects of bioenhancers, such as their detailed modes of action, are still insufficiently addressed. Interference with metabolizing enzymes or multidrug resistance proteins, which limit bioavailability, has been discussed [17]. Importantly, increased absorption by bioenhancers is not limited to nutritional compounds but is also relevant to anticancer and antimicrobial agents to achieve a lower drug dosage [18]. Furthermore, whether lipid-based nanoparticles synergize with phytochemical bioenhancers to provide superior bioavailability remains elusive.

The low bioavailability of compounds can be circumvented by administration via the buccal mucosa. The buccal mucosa is part of the oral mucosa and lines the cheeks, upper and lower inner lips, and gums [19]. It is composed of a stratified squamous nonkeratinous epithelium. The absence of keratin ensures elasticity and enables the entry of compounds into the systemic circulation [20]. Over the past few decades, several studies have focused on the buccal delivery of drugs, including nanoparticle delivery [21]; however, to the best of our knowledge, no study has focused on the delivery of fat-soluble vitamins via buccal nanoparticles.

Enterocytes possess all the machinery necessary for vitamin metabolism, as even inactive forms of vitamins D and K produce biological effects on enterocytes [22,23]. The two forms of vitamin D, vitamin D3 (cholecalciferol) and vitamin D2 (ergocalciferol), are both metabolized to their 1α,25-dihydroxylated forms, i.e., 1α,25-dihydroxyvitamin D3 (1,25(OH)_2_D_3_) or 1α,25-dihydroxyvitamin D2 (1,25(OH)_2_D_2_), to bind to the vitamin D receptor (VDR) [24]. Metabolization involves a two-step process of 25-hydroxylation via CYP2R1 and 1α-hydroxylation via CYP27B1. In contrast to vitamin D2, vitamin D3 can also be 25-hydroxylated by the enzyme, CYP27A1 [25,26,27].

Like vitamins D3 and D2, plant-derived vitamin K1 (phylloquinones) and bacteria-derived vitamin K2 (menaquinones) are not biologically active. In tissues, these precursors are reduced to vitamin K hydroquinone, which acts as a cofactor for gamma glutamyl carboxylase, activating specific proteins, including coagulation factors [28]. In contrast, dietary vitamin E isoforms, i.e., α-, β-, γ-, and δ-tocopherols and tocotrienols, can directly act as antioxidants or regulate gene expression [29]. As a supplement, vitamin E is predominantly provided in the form of an acetyl ester and needs to be hydrolyzed in situ to become bioactive [30].

Vitamins D3 and D2 were previously considered equipotent to cure nutritional rickets [31,32]; however, their equipotency was recently questioned [33,34,35]. Yet, studies comparing the physiological roles of vitamins D3 and D2 are scarce [36]. A recent study comparing the human blood transcriptome after supplementation with vitamin D3 or D2 in the winter provides evidence that vitamin D3 and D2 have both matching and differing biological effects [37], suggesting high complexity related to the equipotency of vitamins D3 and D2 and the need for further exploration.

In this in vitro study, we evaluated whether the coincubation of micellated fat-soluble vitamins D, E (α-tocopheryl acetate), and K2 (menaquinone-7) with the curcuma (*Curcuma longa*), black pepper (*Piper nigrum*), or ginger (*Zingiber officinale Roscoe*) extracts increases the cellular vitamin uptake into the enterocyte model cell line Caco-2. Moreover, the buccal absorption of micellated vitamins was assessed in TR146 cells. Cellular uptake tests were extended via bioefficacy testing to validate the functional relevance of increased vitamin absorption. Finally, the potential differences in the bioefficacy of vitamins D3 and D2 were investigated.

## 2. Materials and Methods

### 2.1. Vitamins and Extracts

Vitamin D3 (1.0 MIU/g, medium-chain triglyceride oil as a carrier, stabilized with ≤1% DL-alpha-tocopherol) and vitamin E acetate (DL-alpha-tocopheryl acetate, ≥98% purity) were obtained from BASF SE (Ludwigshafen, Germany). Vitamin D2 (1.0 MIU/g, sunflower oil as a carrier, stabilized with ≤3% all-rac-alpha-tocopherol) was acquired from Divi’s Laboratories Ltd. (Hyderabad, India). Vitamin K2 (5% all-trans menaquinone-7, with medium-chain triglyceride oil as the carrier) was obtained from Kappa Bioscience AS (Oslo, Norway).

The curcuma extract (CuE), an extract of the dried rhizome of *Curcuma longa* that is complexed with γ-cyclodextrin, was obtained from Wacker Chemie AG (Munich, Germany) and contained 167.56 mg/g curcumin, 39.28 mg/g demethoxy-curcumin, and 5.18 mg/g bisdemethoxy-curcumin, as determined by HPLC-FLD (see the Supplementary Materials). The black pepper extract (BPE), an ethanol extract of *Piper nigrum* fruits from India, was obtained from Sabinsa Europe GmbH (Langen, Germany) and contained 941.9 mg/g piperine, as determined via HPLC-DAD. The ginger extract (GiE), a CO_2_ extract from the dried rhizome of *Zingiber officinale Roscoe,* was obtained from FLAVEX Naturextrakte GmbH (Rehlingen, Germany) and consisted of 30–50% essential oil and 24–35% pungent compounds, predominantly gingerols, according to the manufacturer. The most abundant volatile compounds were zingiberene, β-sesquiphellandrene, β-bisabolene, and α-farnesene, as determined by GC–MS (Supplementary Materials, Table S1). Additionally, gingerols and related compounds were tentatively characterized via HPLC-MS (Supplementary Materials, Table S2).

Both the vitamins and extracts were either tested in pure (“nonmicellated”) or emulsified (“micellated”) forms, as previously described [38]. In more detail, the samples were dissolved in glycerol/water (70:30) with 2% soy lecithin and processed in a high-pressure microfluidizer (5 cycles at 1000 bar; Microfluidics, Westwood, MA, USA). The vitamin concentrations after micellization were confirmed via HPLC-MS. Vitamins in both nonmicellated and micellated forms, as well as the GiE, were not cytotoxic (i.e., cell viability ≥ 90%) at the concentrations tested (Supplementary Materials, Figure S1). The BPE and CuE were likewise not cytotoxic, as determined previously [14].

### 2.2. Cell Culture

Caco-2 cells were obtained from DSMZ (Braunschweig, Germany) and cultivated in Eagle’s minimal essential medium (MEM) supplemented with 10% FBS and 1% penicillin/streptomycin (P/S; all from PAN Biotech GmbH, Aidenbach, Germany) at 37 °C in a humidified 5% CO_2_ atmosphere. The cells were passaged every three to four days using trypsin-mediated detachment.

Human buccal TR146 cells [39] were kindly provided by Dr. Eva Roblegg (University of Graz, Graz, Austria) and maintained in the same way as Caco-2 cells, except that Dulbecco’s modified Eagle’s medium (DMEM; PAN Biotech GmbH) was used.

### 2.3. Cell Seeding, Differentiation, and Treatment

Caco-2 or TR146 cells were seeded into 6-well plates (Caco-2: 2 × 10^6^ cells per well; TR146: 1.5 × 10^6^ cells per well) or 96-well plates (Caco-2: 1.5 × 10^5^ cells per well, TR146: 2 × 10^4^ cells per well) on Day 0. TR146 cells were treated with the respective test solutions on Day 1. Caco-2 cells were differentiated using DMEM supplemented with 5 mM butyric acid and 0.1% serum extender (Corning, NY, USA) on Days 1 and 2, as previously described [40]; test solutions were added on Day 3.

For the treatment, the cells were washed once with PBS and incubated with nonmicellated or micellated preparations or vitamin–extract combinations at the indicated concentrations. Specifically, nonmicellated vitamins were diluted from ethanol stock solutions (final ethanol concentrations were <0.1%, except for vitamin K2, where equivalent ethanol concentrations were added to the controls). Micellated vitamins were directly diluted into the treatment media. The final vitamin concentrations in the treatment media were confirmed via HPLC-MS. The extracts were prepared in ethanol (for the BPE and GiE) or ethanol/FaSSIF (fasted state-simulated intestinal fluid; Klein [41], 60/40, *v*/*v*, for CuE) prior to dilution in the cell treatment medium. The same concentrations (CuE: 14.7 µg/mL; BPE: 6 µg/mL; GiE: 58.8 µg/mL) were used as previously determined [14]. The BPE and GiE were tested in both micellated and nonmicellated forms.

### 2.4. Vitamin Uptake Studies

The cells were seeded into 6-well plates and treated for 4 h with the test substances (i.e., vitamins alone or in combination with plant extracts) diluted in HBSS containing 5 mg/mL bovine serum albumin (BSA), as described above. In addition, cell-free wells containing the same test solutions were included to correct for the potential adhesion of the vitamins to the plastic surface. After 4 h of incubation, the cells were washed twice with ice-cold HBSS containing 5 mg/mL BSA. The vitamins were extracted from the cell layers twice with 1 mL of hexane/isopropanol (3:2, *v*/*v*) for 10 min. The extractions were combined and the solvents were evaporated using a centrifugal vacuum concentrator (40–45 °C). The vitamins were redissolved in 50% isopropanol and 1% formic acid (500 µL), and their concentrations were determined via HPLC-MS analysis. The dry cell layers were lysed with 0.1 M NaOH (2 mL) for 1 h to subsequently determine the protein content per well via the Bradford assay (Bio-Rad Laboratories, Hercules, CA, USA) according to the manufacturer’s instructions. The vitamin uptake was corrected for cell-free wells and normalized to the protein content of the cells. In the case of vitamin E, the data were first transformed into nmol of vit/mg of protein before the relative uptake ratio was calculated.

### 2.5. HPLC–MS Analyses

Cholecalciferol, ergocalciferol, calcidiol, α-tocopheryl acetate, free α-tocopherol, menaquinone-7, and menaquinol-7 were quantified via LC-MS employing an external standard calibration method. A Vanquish Flex UHPLC system equipped with a built-in degasser, a binary pump, an autosampler, a heated column compartment, and an ISQ-EC mass spectrometer with an HESI ion source (all Thermo Fisher Scientific, Waltham, MA, USA) was used. Chromatographic separation was achieved using an Accucore C18 column (150 mm × 2.1 mm inner diameter, 2.6 µm particle size; Thermo Scientific) heated to 60 °C with an injection volume of 4 µL. Gradient elution was performed at a flow rate of 0.5 mL/min with mobile phases A (50% isopropanol and 5 mM ammonium formate, pH 3.65) and B (isopropanol and 5 mM ammonium formate, pH 3.65), starting at 99.9% A and 0.1% B, then increasing to 99.9% B within 5 min and holding for 2 min at 99.9% B. Finally, the amount of mobile phase B was reduced to 0.1% again, and the column was equilibrated for 3 min prior to the next injection. The mass spectrometer was operated in the positive ion mode, and the HESI source was set to a voltage of 3.0 kV, with the vaporizer heated to 280 °C and the transfer tube heated to 250 °C. The sheath gas pressure was set to 47.3 psig, the auxiliary gas pressure was set to 8 psig, and the sweep gas pressure was set to 1 psig. The spectra were recorded in the single ion monitoring (SIM) mode with the isolation width set to 0.2 *m*/*z* and a dwell time of 0.1 s per scan. The monitored ions are detailed in Table 1. Instrument operation and data analysis were performed using the Chromeleon 7.2 software package (Thermo Scientific, Waltham, MA, USA), and for quantification, pure reference compounds (all obtained from Sigma–Aldrich, St. Louis, MO, USA, purity ≥ 96% determined via HPLC) were used, a five-point calibration curve was produced, and a linear regression analysis was performed using the software (R^2^ ≥ 0.95).

### 2.6. Gene Expression Analysis

The cells were seeded into 6-well plates and treated for 24 h with the test substances (i.e., vitamins alone or in combination with plant extracts) in Eagle’s MEM containing 1% P/S and 5 mg/mL BSA, as described above. RNA was isolated using the RNeasy^®^ Plus Mini Kit (QIAGEN, Hilden, Germany) in combination with Precellys^®^ MK28 hard tissue grinding tubes (Bertin Technologies SAS, Montigny-le-Bretonneux, France). In more detail, 350 µL of cell lysate (lysis buffer from the RNeasy^®^ Plus Mini Kit) was centrifuged in Precellys^®^ tubes (Precellys^®^ Evolution homogenizer: 6000 rpm, 15 s × 2, pause: 1 min on ice), followed by RNA isolation. Next, RNA was transcribed into cDNA via the iScript^TM^ cDNA Synthesis Kit (Bio-Rad Laboratories). Subsequently, reverse transcription qPCR (RT–qPCR) was performed in a singleplex assay using iQ^TM^ SYBR^®^ Green Supermix and the C1000 Touch^TM^ thermal cycler in combination with the CFX96 ^TM^ Real-Time PCR System or the CFX Connect^TM^ Real-Time PCR System (all from Bio-Rad Laboratories). PCR was performed at an annealing temperature of 60 °C, and the primer sequences can be found in Supplementary Table S3. The target gene expression was normalized to that of the housekeeping genes, *HPRT1* and *GAPDH*. The relative mRNA expression was calculated by using the $2^{-∆∆C_{T}}$ (Livak) method.

### 2.7. NO Assay

The cells were seeded into 96-well plates and treated for 24 h with the test substances (i.e., vitamins alone or in combination with plant extracts) in phenol red-free MEM Eagle (PAN-Biotech GmbH) containing 1% P/S and 5 mg/mL BSA, as described above. Subsequently, the content of nitric oxide (NO) in the fresh supernatant was measured indirectly via its breakdown product, nitrite, using a Griess assay kit (Promega, Madison, WI, USA).

### 2.8. Statistical Analysis

The data are shown as the means ± SDs. Statistical analyses were performed using Graph Pad Prism (Graph Pad Software, San Diego, CA, USA; ver. 9.5.1). Each dataset was tested for a normal distribution using the Shapiro–Wilk test. For the analysis of normally distributed data, a *t* test with Welch’s correction was performed to compare two groups, whereas one-way ANOVA was performed for comparisons of more than two groups; Dunn’s post hoc test was used for comparisons against the control, and the Šidák test was used for comparisons of two groups not involving the control. Nonparametric data were compared using the Mann–Whitney test (two groups). If the latter was the case, it is mentioned in the figure legends. *p* values indicating statistical significance are denoted as * *p* ≤ 0.05, ** *p* ≤ 0.01, *** *p* ≤ 0.001 and **** *p* ≤ 0.0001. “ns” indicates no statistical significance.

## 3. Results

### 3.1. Micellization Increases the Uptake of Vitamins D3 and K2 into Buccal and Intestinal Cells and of Vitamin E into Intestinal Cells

Uptake experiments in two immortalized human cell lines, i.e., buccal TR146 cells and differentiated intestinal Caco-2 cells, were conducted to investigate whether micellization increases the absorption of vitamin D3, vitamin E acetate, and vitamin K2. Differentiated Caco-2 cells resemble small intestinal epithelial cells in terms of function, morphology, and the expression of digestive enzymes and active membrane transporters [42], whereas TR146 cells are used for studies of the oral buccal mucosa [43].

**Figure 1:**
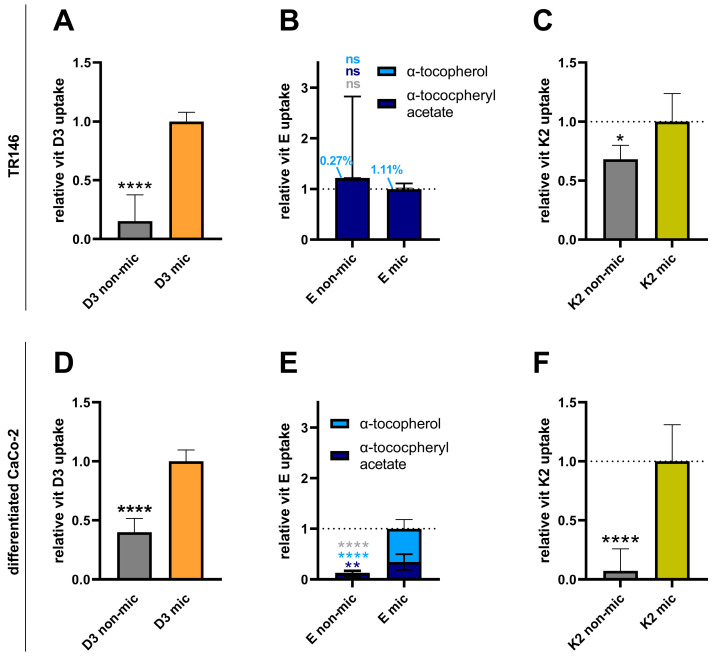
Effect of micellization on the buccal and intestinal uptake of vitamins D3, E, and K2. TR146 (**A**–**C**) or differentiated Caco-2 cells (**D**–**F**) were treated with vitamins (vitamin D3, 3.9 µM; vitamin E acetate, 3 µM; vitamin K2, 2.3 µM) in either nonmicellated (“non-mic”) or micellated forms (“mic”) for 4 h. Afterward, vitamins were extracted and quantified via HPLC–MS analysis. The data were derived from two (**A**,**C**–**F**) or four (**B**) independent experiments performed in triplicate or quadruplicate. (**B**): The Mann–Whitney test was used for the statistical analysis.* *p* ≤ 0.05, ** *p* ≤ 0.01 and **** *p* ≤ 0.0001. “ns” indicates no statistical significance. Significance levels in (**B**,**E**) refer to comparisons the levels of α-tocopheryl acetate (dark blue), α-tocopherol (light blue) and total vitamin E (grey).

Cellular vitamin uptake was determined for the pure vitamin form (denoted as “nonmicellated”) or after the vitamin had undergone micellization (denoted as “micellated”). The micellization of vitamin D3 significantly increased its uptake into buccal TR146 and intestinal Caco-2 cells by ~6.5- and ~2.5-fold, respectively (Figure 1A,D). The presence of the intermediate metabolite 25-hydroxyvitamin D3 (calcidiol) was monitored but was not detected at a lower limit of detection (LOD) of 19 ng/mL.

Vitamin E acetate must undergo hydrolysis upon absorption to form active free alpha tocopherol. Thus, both free vitamin E and vitamin E acetate levels were measured in the uptake experiments. The uptake of vitamin E acetate into buccal TR146 cells was not significantly altered upon micellization (Figure 1B). However, large interexperimental variations were observed for nonmicellated vitamin E acetate. Free vitamin E was not detected in these cells after the uptake experiments. In the intestinal Caco-2 cells, the vitamin E acetate uptake was significantly increased ~9-fold upon micellization, and abundant free vitamin E was detected in the uptake experiments with micellated vitamin E acetate (Figure 1E).

The vitamin K2 uptake into the buccal TR146 and intestinal Caco-2 cells was also significantly increased upon micellization (by 1.5-fold and 14-fold, respectively; Figure 1C,F). The reduced metabolic intermediate menaquinol-7 was not detected (LOD: 113 ng/mL).

### 3.2. Plant Extracts from Curcuma, Black Pepper, and Ginger Specifically Increase the Uptake of Micellated Vitamins

**Figure 2:**
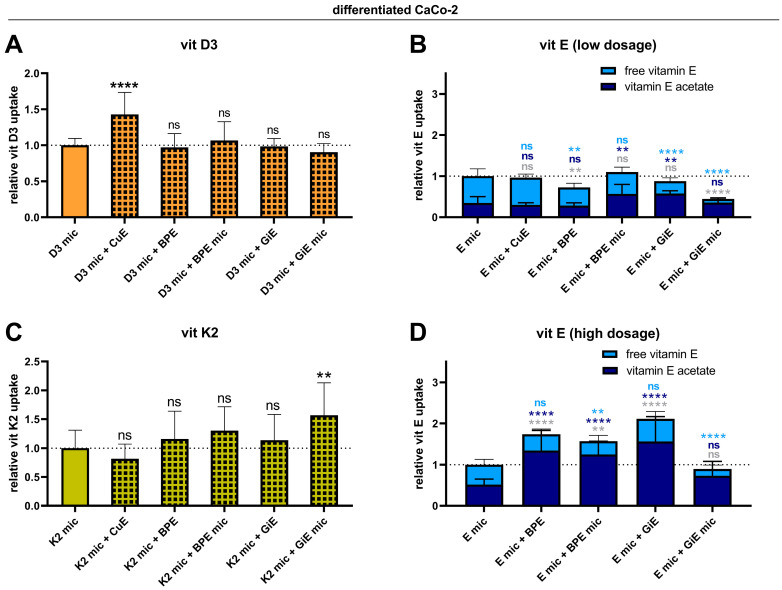
Effects of micellization in combination with plant extracts on the intestinal uptake of vitamins D3, E, and K2. Differentiated Caco-2 cells were either treated with micellated vitamins (vitamin D3, 3.9 µM (**A**); vitamin E acetate, 3 µM (**B**) and 22.5 µM (**D**); or vitamin K2, 2.3 µM (**C**) alone or in combination with CuE (CuE, 14.7 µg/mL), nonmicellated BPE (BPE, 6 µg/mL), micellated BPE (BPE mic, 6 µg/mL), nonmicellated GiE (GiE, 58.8 µg/mL), or micellated GiE (GiE mic, 58.8 µg/mL) for 4 h. Afterward, vitamins were extracted and quantified via HPLC–MS analysis. The data shown were obtained from two to four independent experiments performed in triplicate. ** *p* ≤ 0.01, **** *p* ≤ 0.0001. “ns” indicates no statistical significance. Significance levels in (**B**,**D**) refer to comparisons the levels of α-tocopheryl acetate (dark blue), α-tocopherol (light blue) and total vitamin E (grey).

Selected plant extracts were tested for their potential interactions with intestinal vitamins D3, E, and K2 uptake in differentiated Caco-2 cells to investigate whether plant extracts are capable of increasing the vitamin uptake on top of micellization. The CuE significantly increased the uptake of micellated vitamin D3 by 75%. In contrast, the BPE and GiE had no effects, irrespective of whether the vitamins were micellated (Figure 2A).

The total intracellular vitamin E content (i.e., free vitamin E plus vitamin E acetate) after the uptake experiments was not elevated upon coincubation when vitamin E acetate was administered at a low dose (3 µM, Figure 2B). Instead, the uptake even decreased when this vitamin was applied together with some extracts. In contrast, when higher vitamin E acetate doses were applied (22.5 µM), the BPE in both nonmicellated and micellated forms, as well as the GiE in the nonmicellated form, resulted in a significantly higher intracellular total vitamin E content (Figure 2D). However, no increase in the free vitamin E content was observed. Therefore, we hypothesized that the conversion rate of vitamin E acetate into free vitamin E is diminished by the plant extract applied. Indeed, the carboxylesterase activity in the Caco-2 cells was significantly reduced by the GiE (Supplementary Materials, Figure S2). Similarly, the BPE tended to reduce carboxylesterase activity.

The uptake of vitamin K2 was not increased by the CuE or BPE but was increased by 57% when the GiE was applied in its micellated form (Figure 2C).

### 3.3. Micellization Increases the Bioefficacy of Vitamin D3 but Does Not Synergize with Plant Extracts

An analysis of the target gene expression was conducted in the Caco-2 cells to determine whether the increase in vitamin D3 uptake leads to increased bioefficacy. We detected the robust expression of VDR and the key enzymes necessary for D3/D2 activation (CYP2R1, CYP27A1, and CYP27B1) in a significant number (Supplemental Material, Figure S3) of these cells. Under basal conditions, the enzyme involved in vitamin D3/D2 deactivation (CYP24A1) was barely expressed, which is expected in the absence of excess vitamin D [44].

For vitamin D3, the expression of two genes involved in intestinal calcium absorption was analyzed: (1) *TRPV6* (transient receptor potential channel family, vanilloid subfamily member 6), which mediates the intestinal transcellular uptake of calcium, and (2) *CLDN2,* encoding claudin 2, a channel-forming tight junction protein that regulates paracellular calcium transport. The transcription of both genes is induced upon the binding of 1,25(OH)_2_D to the VDR [45].

**Figure 3:**
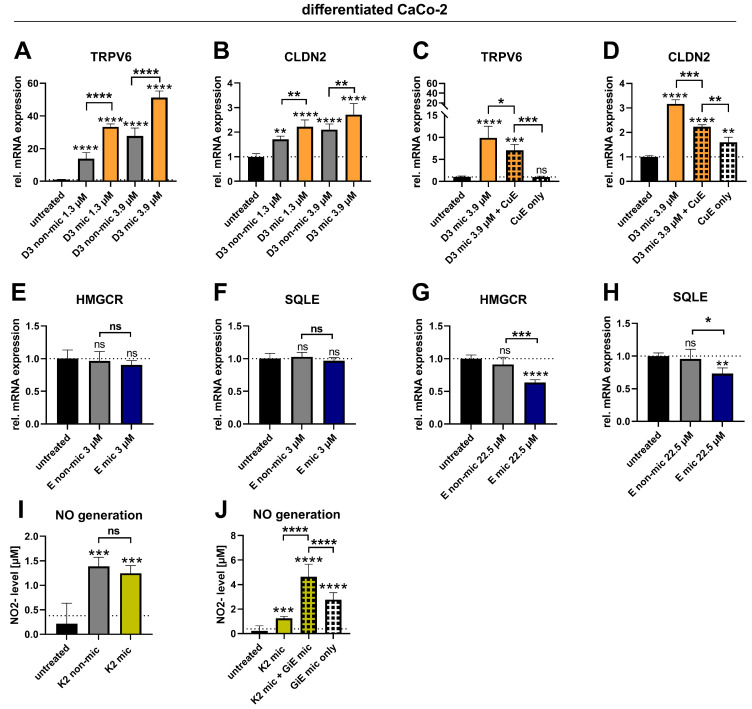
Effects of micellization alone or in combination with plant extracts on biological markers of vitamin D3, E, and K2 function in Caco-2 cells. Differentiated Caco-2 cells were either not treated with the test substances (“untreated”) or treated with the vitamins (vitamin D3, 1.3 µM, 3.9 µM; vitamin E acetate, 3 µM and 22.5 µM; vitamin K2, 2.3 µM) in the nonmicellated form (“non-mic”), micellated form (“mic”), or micellated form in combination with CuE (CuE, 14.7 µg/mL) or micellated GiE (GiE mic, 58.8 µg/mL) for 24 h. Afterward, RNA was extracted and analyzed for TRPV6, CLDN2, HMGCR, or SQLE expression via RT–qPCR (**A**–**H**), or the nitrite concentration in the cell supernatant was measured using the Griess assay (**I**,**J**). The data shown were obtained from two independent experiments performed in duplicate (**A**–**H**) or triplicate (**I**,**J**). * *p* ≤ 0.05, ** *p* ≤ 0.01, *** *p* ≤ 0.001 and **** *p* ≤ 0.0001. “ns” indicates no statistical significance.

In agreement with the uptake results, the gene expression of *TRPV6* and *CLDN2* was significantly increased upon treatment with micellated vitamin D3 compared with nonmicellated vitamin D3 in a dose-dependent manner (TRPV6: *p* ≤ 0.0001 for D3 non-mic vs. D3 mic at both concentrations; CLDN2: *p* = 0.0077 and *p* = 0.0032 for D3 non-mic vs. D3 mic at 1.3 µM and 3.9 µM, respectively; Figure 3A,B). Micelles alone had no effect on the expression of either *TRPV6* or *CLDN2* (Supplemental Material, Figure S4A,B), which confirms that the observed biological effects solely stem from the increased vitamin D3 uptake.

The coincubation of micellated vitamin D3 with the CuE, which increased the vitamin D3 uptake (compare with Figure 2A), unexpectedly and significantly reduced the gene expression of both *TRPV6* and *CLDN2* compared with that of micellated vitamin D3 alone (Figure 3C,D). In addition, the CuE alone slightly increased the *CLDN2* expression but had no effect on the *TRPV6* expression.

### 3.4. Micellated Vitamin E Acetate Reduces the Expression of Genes Involved in Cholesterol Biosynthesis

For vitamin D3, bioefficacy tests of vitamin E were performed to functionally validate the results from the uptake study. The targets selected for vitamin E acetate were two genes encoding the rate-limiting enzymes of cholesterol synthesis, i.e., 3-hydroxy-3-methylglutaryl-coenzyme A reductase (*HMGCR*) and squalene epoxidase (*SQLE*). Alpha tocopherol was previously shown to reduce the expression of these genes in the Caco-2 cells [46]. Notably, the cholesterol synthesis within enterocytes is a significant contributor to the plasma cholesterol pool [47].

The gene expression of *HMGCR* and *SQLE* was not altered by low vitamin E acetate concentrations (3 µM), irrespective of micellization (Figure 3E,F). However, micellization reduced the expression of both genes (by ~40% for *HMGCR* and ~30% for *SQLE*) when applied at pharmacologically relevant concentrations (22.5 µM; Figure 3G,H), consistent with the observation that only micellated vitamin E acetate increased the cellular level of free vitamin E. Micelles containing no vitamin E acetate did not alter the expression of *HMGCR* or *SQLE* (Supplemental Material, Figure S4C,D). Plant extracts were not tested in addition to micellization, since no increase in free vitamin E levels was observed in the uptake experiments.

### 3.5. The Ginger Extract Increases NO Production in Addition to Vitamin K2

Next, the bioefficacy of vitamin K2 was tested to functionally validate the consequences of increased vitamin K2 uptake due to micellization and coincubation with the GiE. Nitric oxide (NO) production was measured because (i) vitamin K2 alleviates intestinal inflammation [3], (ii) the formation of NO is induced by vitamin K2 in other tissues [48], and (iii) the NO production in enterocytes is relevant to the amelioration of colitis [49].

As hypothesized, the NO concentrations were increased in the vitamin K2-treated Caco-2 cells compared to the untreated cells (Figure 3I). Despite increasing the vitamin K2 uptake, micellization did not further increase the NO generation. The combination of micellated vitamin K2 with the micellated GiE, which increased the vitamin K2 uptake, likewise increased the NO generation to a greater extent than micellated vitamin K2 alone. However, this effect was additive rather than synergistic because the micellated GiE alone also increased the NO production (Figure 3J). Importantly, micelles containing neither vitamin K2 nor the GiE induced NO production (Supplemental Material, Figure S4E).

### 3.6. Vitamin D3 Is More Potent than Vitamin D2 in Upregulating the Genes Involved in Intestinal Calcium Absorption

**Figure 4:**
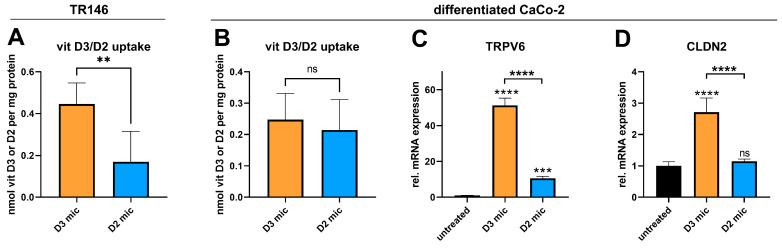
Comparison of vitamin D3 and vitamin D2 in terms of their buccal and intestinal absorption efficiencies and effects on intestinal calcium absorption markers. TR146 (**A**) or differentiated Caco-2 cells (**B**–**D**) were either untreated or treated with micellated vitamin D3 (D3 mic; 3.9 µM) or micellated vitamin D2 (D2 mic; 3.9 µM) for 4 h (**A**,**B**) or 24 h (**C**,**D**). After 4 h of treatment, vitamins D3 and D2 were extracted and quantified via HPLC–MS analysis. After 24 h of treatment, RNA was extracted and analyzed for TRPV6 or CLDN2 expression via RT–qPCR (**C**,**D**). The data were obtained from two independent experiments performed in quadruplicate (**A**), five independent experiments performed in triplicate (**B**), or two independent experiments performed in duplicate (**C**,**D**). ** *p* ≤ 0.01, *** *p* ≤ 0.001 and **** *p* ≤ 0.0001. “ns” indicates no statistical significance.

Finally, we analyzed whether the uptake of vitamins D3 and D2 in buccal and intestinal cells was comparable and whether they were equipotent in inducing the expression of genes that regulate intestinal calcium absorption. In buccal cells, micellated vitamin D2 was taken up less efficiently than vitamin D3 was (Figure 4A). In contrast, their uptake into the Caco-2 cells was comparable (Figure 4B). Despite comparable uptake rates, vitamin D3 more potently induced the expression of the VDR-regulated genes *TRPV6* (51-fold vs. 11-fold) and *CLDN2* (2.7-fold vs. 1.1-fold; Figure 4C,D).

**Figure 5:**
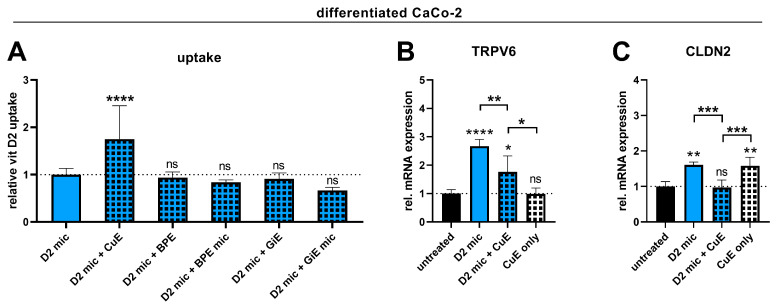
Effect of micellization in combination with plant extracts on intestinal vitamin D2 uptake and on biological markers of vitamin D function in Caco-2 cells. Differentiated Caco-2 cells were either untreated or treated with micellated vitamin D2 (D2 mic, 3.9 µM) alone or in combination with CuE (CuE, 14.7 µg/mL), nonmicellated BPE (BPE, 6 µg/mL), micellated BPE (BPE mic, 6 µg/mL), nonmicellated GiE (GiE, 58.8 µg/mL), or micellated GiE (GiE mic, 58.8 µg/mL) for 4 h (**A**) or with D2 mic and/or CuE for 24 h (**B**,**C**). After 4 h of treatment, vitamin D2 was extracted and quantified via HPLC–MS analysis. After 24 h of treatment, RNA was extracted and analyzed for TRPV6 and CLDN2 expression via RT–qPCR. The data shown were obtained from two to four independent experiments performed in triplicate (**A**) or two independent experiments performed in duplicate (**B**,**C**). * *p* ≤ 0.05, ** *p* ≤ 0.01, *** *p* ≤ 0.001 and **** *p* ≤ 0.0001. “ns” indicates no statistical significance.

The experiments assessing vitamin D2 uptake and bioefficacy were conducted in the same way as those involving vitamin D3 to test whether the vitamin D2 uptake was affected by the plant extracts in a manner similar to that of vitamin D3. In contrast, only the CuE increased the uptake of vitamin D2 into differentiated Caco-2 cells, whereas the BPE and GiE in both the micellated and nonmicellated forms had no effect (Figure 5A). Despite increasing the vitamin D2 uptake, the CuE decreased the vitamin D2-induced expression of the *TRPV6* and *CLDN2* genes (Figure 5B,C), indicating that the CuE affects the uptake and bioefficacy of both vitamins D2 and D3 in a similar manner.

## 4. Discussion

Here, we investigated how selected plant extracts affect the in vitro buccal and intestinal absorption and bioefficacy of fat-soluble vitamins on top of micellization. We showed that micellization increased the uptake of vitamin D3, vitamin E acetate, and vitamin K2 in human intestinal and buccal cell models. The addition of selected plant extracts to micellated vitamins further increased the vitamin uptake into intestinal cells. However, this effect was vitamin specific instead of universal. A positive effect was observed for the curcuma extract (CuE) on the vitamin D3 and D2 uptake and for the micellated GiE on the vitamin K2 uptake. With respect to the vitamin E acetate uptake, the micellated and nonmicellated black pepper extract (BPE) and nonmicellated ginger extract (GiE) had positive effects. However, this effect was restricted to a higher vitamin E acetate concentration of 22.5 µM, which was still within the range of the human plasma vitamin E levels. Moreover, we showed that vitamins D3 and D2 differed in their ability to upregulate the expression of the genes involved in intestinal calcium absorption, with vitamin D3 being more potent.

While a comprehensive understanding of the detailed mechanisms mediating the absorption of fat-soluble vitamins remains elusive, several overlapping uptake mechanisms in intestinal cells have been identified. Receptors such as scavenger receptor class B type I (SR-BI), Niemann–Pick C1-like 1 (NPC1L1), and cluster of differentiation 36 (CD36) are classically known as cholesterol transporters, possess a large spectrum of ligands, and are involved in the uptake of vitamins D, E, and K [50]. As the plant extracts CuE, GiE, and BPE induced the uptake in a vitamin-specific manner, it is unlikely that they act via these common vitamin transporters. The BPE, for example, solely altered the uptake of vitamin E but not that of vitamins D3, D2, or K2. The CuE, on the other hand, solely increased the uptake of vitamin D. The latter effect is possibly mediated by the curcumin-mediated inhibition of P-glycoprotein [51], which is involved in the efflux of absorbed vitamin D back into the intestinal lumen [52]. However, the detailed modes of action for each of the plant extracts remain to be investigated.

Throughout this study, we observed that the increased intestinal vitamin uptake itself does not necessarily encompass increased bioefficacy. While the CuE further increased the uptake of vitamin D3 in addition to micellization, this effect did not translate into the increased expression of the genes involved in intestinal calcium uptake. Rather, the expression of the VDR target genes, *TRPV6* and *CLDN2*, was repressed in the presence of the CuE. Interestingly, curcumin, a major constituent of curcuma, competes with 1,25(OH)_2_D_3_ for binding to the VDR and is a less potent activator than 1,25(OH)_2_D_3_ itself [53]. This result might explain why the increased vitamin D3 uptake in the presence of the CuE does not increase the expression of the VDR target genes.

Similarly, an increase in the vitamin E acetate uptake in addition to micellization by the plant extracts did not result in an increase in the amount of biologically active free vitamin E. Rather, the intracellular ratio between free vitamin E and vitamin E acetate was shifted toward inactive vitamin E acetate by the BPE and GiE. Like the intestine, Caco-2 cells express carboxylesterases [54], which catalyze vitamin E acetate hydrolysis [55]. Here, we showed that the GiE inhibited carboxylesterase activity, consistent with other studies using isolated, recombinant carboxylesterase-1 [56]. Moreover, the BPE tended to inhibit carboxylesterase activity, which together might explain the lack of increase in the amount of biologically active free vitamin E induced by the plant extracts despite the increase in the total vitamin E content.

While the conversion of vitamin E acetate into free vitamin E was abundant in the Caco-2 cells, it was barely detected in the buccal TR146 cells. In contrast, the hydrolysis of vitamin E acetate was detected in the reconstituted human buccal epithelium [57]. In this study, the vitamin concentrations used were four orders of magnitude higher, and the incubation times ranged from up to seven days. These findings indicate that the rate of vitamin E acetate hydrolysis is limited in buccal cells.

Our results further suggested that vitamin K increased the NO levels in Caco-2 cells, which has already been described for the endothelium [48]. Here, the concomitant application of the GiE, which increased the vitamin K2 uptake, further increased the NO production. However, the effect of the GiE was likely additive and not based on the increased uptake of vitamin K2. This result is because the GiE alone likewise induced NO levels. Presumably, the effect of vitamin K on the NO production is saturable because the increased uptake due to micellization also does not increase the NO production. Notably, the effect of the GiE on the NO levels is consistent with reports on the vasodilatory effects of ginger compounds [58,59,60].

Finally, vitamins D3 and D2 were compared in terms of their cellular uptake, bioefficacy, and interaction with the plant extracts. This analysis is relevant since the demand for vitamin D2 supplements derived from nonanimal sources is increasing. While vitamin D3 and vitamin D2 exhibited similar uptakes and interactions with the plant extracts in the Caco-2 cells, vitamin D3 was more potent in increasing the upregulation of *TRPV6* and *CLDN2*. This finding is consistent with studies reporting increased calcium transport by vitamin D3 compared with vitamin D2 [61] and a meta-analysis showing that vitamin D3 is more efficient in increasing the vitamin D levels in humans [35]. These differences in potency need to be considered when vitamin D2 is preferred over D3 as a supplement.

## 5. Conclusions

In summary, our data further substantiate the positive effects of micellization on fat-soluble vitamin absorption and bioefficacy. Our experimental study focused solely on buccal and intestinal cellular absorption, which is a limitation of our study. In particular, the influences of digestive enzymes, bile acids, and changes in the intestinal pH, which have been shown to influence vitamin absorption [62], could not be considered. Despite these limitations, the studies of humans confirm that micellated formulations of fat-soluble vitamins are safe and efficient [63,64]. The combination of micellated vitamins with plant extracts to further increase the efficiency is an interesting approach; however, further studies are warranted because our data show that an increased uptake is not necessarily accompanied by increased bioefficacy. Finally, our study suggests that the buccal delivery of vitamins is a putative strategy to overcome the vitamin deficiencies in patients with impaired intestinal absorption.

## Figures and Tables

**Table 1:** Summary of all monitored ions and the respective applied in-source CID voltages.

Compound	Monitored Ion (*m*/*z*)	Species	In-Source CID Voltage
α-tocopheryl acetate	490.5	[M + NH_4_]^+^	0
	207.2	[M + H]^+^	70
	165.2	[M + H]^+^	80
	137.2	[M + H]^+^	90
α-tocopherol	431.5	[M + H]^+^	0
	165.2	[M + H]^+^	80
	137.2	[M + H]^+^	90
ergocalciferol	397.3	[M + H]^+^	0
	133	[M + H]^+^	60
	107	[M + H]^+^	60
cholecalciferol	385.5	[M + H]^+^	0
	107	[M + H]^+^	60
	91	[M + H]^+^	90
calcidiol	401.5	[M + H]+	0
	107	[M + H]+	60
	91	[M + H]+	90
menaquinone-7	666.7	[M + NH_4_]^+^	0
menaquinol-7	668.7	[M + NH_4_]^+^	0

## Data Availability

The original contributions presented in this study are included in the article and Supplementary Materials. Further inquiries can be directed to the corresponding authors.

## Supplementary Materials

The following supporting information can be downloaded at: https://www.mdpi.com/article/10.3390/nu17020359/s1, Figure S1: Cytotoxicity testing of vitamins and extracts used in uptake and bioefficacy studies; Figure S2: Effects of black pepper extract and ginger extract on carboxylesterase activity; Figure S3: Validation of Caco-2 cells as model for vitamin D3/D2 effects; Figure S4: Effects of micelles alone on biological targets; Table S1: Volatile compounds in ginger extract identified via GC analysis; Table S2: Gingerol-related compounds in ginger extract identified via HPLC-MS analysis; Table S3: List of Homo sapiens primers used in RT-qPCR experiments. References [14,65,66,67] are cited in the Supplementary Materials.
