# Supplementary material for: Differential Enhancement of Fat-Soluble Vitamin Absorption and Bioefficacy via Micellization in Combination with Selected Plant Extracts In Vitro
Source: Nutrients. 2025 Jan 20;17(2):359. doi: 10.3390/nu17020359 (PMC11769215; doi:10.3390/nu17020359)
Supplement: Supplementary file 1 [file nutrients-17-00359-s001.zip › nutrients-3398652-supplementary.pdf]

Supplementary material to

Differential enhancement of fat-soluble vitamin absorption and bioefficacy by micellization in combination with selected plant extracts *in-vitro*

Stefanie Steinbauer et al.

1 Supplementary methods

1.1 Extract characterization

Curcuma extract (CuE) and black pepper extract (BPE) were characterized as described previously [1]. In brief, extracts were dissolved in ethanol, diluted in ultrapure water and analyzed by HPLC-FLD and HPLC-DAD, respectively. In both cases, an Ultimate 3000 HPLC System equipped with an Accucore C18 column (150 x 3 mm, 2.6 µm particle size, both Thermo Fisher Scientific, Bremen, Germany) and 0.1% formic acid (FA) in water as solvent A and 0.1% FA in acetonitrile as solvent B was used. For detection of curcuminoids, the FLD wavelengths were set to 432 and 535 nm for excitation and emission, respectively. For BPE analysis, UV signals were recorded at 249 nm. Reference standards for curcumin, demethoxycurcumin and bisdemethoxycurcumin were obtained from Extrasynthese (Lyon, France). Piperine standard was purchased from Sigma Aldrich (St. Louis, LO, USA) and quantification of these compounds was carried out by external standard calibration using a linear regression model ($R^2 \geq 0.95$).

For analysis of the ginger extract (GiE) both GC-MS and LC-MS methods were applied. GC-MS analysis was carried out using a Trace 1300 gas chromatograph coupled to an ISQ QD single-quadrupole mass spectrometer equipped with a PTV injector and a TriPlusRSH autosampler (all Thermo Scientific, Waltham, MA, USA). Injector temperature was kept constant at 240°C and 0.5 µL of the sample diluted in n-hexane was injected onto a Stabilwax-DA column (30 m × 0.25 mm, 0.25 µm film thickness; Restek, Centre County, PA, USA) using a split-ratio of 1:20. Helium was used as carrier gas at a constant flow rate of 1.0 mL/min. Oven temperature was kept constant at 45°C for 5 min, then raised to 240°C at 7 °C/min, followed by a constant period of 10 min at 240°C before returning to start conditions. The injector and ion source temperatures were set to 250°C and 240°C, respectively. Full scans from m/z 40-550 were recorded with a rate of 5 scans/s. Instrument operation and data analysis were performed using the Chromeleon 7.2 software package (Thermo Scientific, Waltham, MA, USA) and analytes were tentatively identified using the NIST 11 spectral library in conjunction with the NIST 11 GC RI database.

For LC-MS analysis, a Vanquish Flex UHPLC system was used, equipped with a built-in degasser, a binary pump, autosampler, heated column-compartment, coupled to an ISQ-EC mass spectrometer via a HESI ion-source (all Thermo Fisher Scientific, Waltham, MA, USA). Chromatographic separation was achieved using an Accucore C18 column (150 mm x 2.1 mm inner diameter, 2.6 µm particle size, Thermo Scientific) heated to 30 °C and an injection volume of 4 µL. Gradient elution was performed at a flow rate of 0.3 mL/min with mobile phase A (5 mM ammonium formate, pH 3.65) and B (90% acetonitrile, 5 mM ammonium formate, pH 3.65), starting at 95% A and 5% B, then increasing to 40% B within 4 minutes and to 99.9%B within further 16 min, followed by a hold-time of 6 minutes at 99.9% B. Finally, B was reduced to 5% again and the column was equilibrated for 5 minutes prior to the next injection. The mass spectrometer was operated in positive ion mode and the HESI source was set to a voltage of 3.5 kV with the vaporizer heated to 350 °C and the transfer tube to 300 °C. Sheath gas pressure was set to 50 psig, Aux gas to 15 psig and Sweep gas to 1 psig. Spectra were recorded in scan mode in a range from 175 to 600 m/z with a rate of 5 scans/s. Multiple runs were recorded with increasing in-source fragmentation voltage from 0 to 100 V. Compounds were tentatively identified by comparison of the molecular and fragment ion masses to public spectral databases and literature [2,3].

1.2 Cytotoxicity testing

Cells were seeded into 96-well plates and treated with the test substances as described in section 2.3. in the main manuscript. After a 4 h or 24 h incubation period, medium was removed, 1 mM resazurin sodium salt (Sigma-Aldrich) diluted 1:20 in full growth medium was added to cells and cells were then incubated for at 37 °C for 90 min. Conversion of resazurin into resorufin was measures fluoreometrically (Ex: 544, Em: 590).

1.3 Carboxylesterase activity assay

The carboxylesterase-activity measurement was based on the formation of the fluorescent substance 4-MUB (4-methylumbelliferone) upon the cleavage of the 4-MUBA-substrate (4-methylumbelliferyl acetate) by carboxylases (CES) from CaCo-2 cells. Analyses were performed from cell lysate as described by Lamego et al. [4], with slight modifications. CaCo-2 cells ($1.5*10^5$ cells per well) were seeded in 96-well plates and grown for 24 h and differentiated as described in the main manuscript. On day 3, cells were lysed on ice.

BNPP (bis(4-nitrophenyl) phosphate; Sigma-Aldrich), an inhibitor of hCES1 and hCES2, was used as positive control. Extracts were prepared as described in the main manuscript, except that FaSSIF was omitted.

Each reaction contained either 50 μL cell lysate or 50 μL lysis buffer (blanks) and 0.05 mM MUBA (Sigma-Alrich) without or with extracts or BNPP in phosphate buffer. Standard reference curves were included using 0 - 75 μM 4-MUB (Sigma-Aldrich) in lysis buffer. Samples were incubated for at 37 °C for 10 min, subsequently 4-MUB signal was measured at 37 °C every 60 s (Ex: 355 nm, Em: 460 nm). Blanks were subtracted from test wells to correct for spontaneous 4-MUBA hydrolysis and background signal of the extracts/BNPP themselves. Within the period of linear enzymatic reaction, data were evaluated as 4-MUB-formation per min.

2 Supplementary tables

Table S1. Volatile compounds in ginger extract identified by GC analysis.

	**retention time**	**measured retention index**	**theor. retention index**	**NIST library search prob.**	**relative peak area (%)**	**area**
**compound**	min	Kováts RI	Kováts RI	%	%	counts*min
Camphene	6.188	1066.6	1074.0	63.0	1.76	$3.41*10^6$
3,4-Dimethyl-1,5-cyclooctadiene	9.837	1198.9	-	24.2	0.48	$9.38*10^5$
Cineole	10	1205.5	1209.0	93.0	1.51	$2.92*10^6$
ß-Phellandrene	10.058	1207.8	1212.0	51.1	1.9	$3.69*10^6$
α-Copaene	16.214	1497.4	1493.0	30.9	0.43	$8.42*10^5$
ß-Linalool	17.2	1551.2	1555.0	88.1	0.3	$5.90*10^5$
L-Borneol	19.904	1709.2	1704.0	51.0	1.2	$2.33*10^6$
Zingiberene	20.224	1728.9	1728.0	70.6	30.63	$5.94*10^7$
ß-Bisabolene	20.319	1734.8	1736.0	42.5	6.87	$1.33*10^7$
α-Farnesene	20.646	1755.2	1753.0	56.9	6.69	$1.30*10^7$
ß-Sesquiphellandrene	21.027	1779.3	1782.0	60.3	12.4	$2.40*10^7$
ar-Curcumene	21.091	1783.4	1773.0	76.7	7.33	$1.42*10^7$
Geraniol	22.152	1852.2	1850.0	64.9	0.41	$8.02*10^5$
(±)-trans-Nerolidol	24.951	2045.3	2044.0	51.8	0.57	$1.10*10^6$
sesquisabinene hydrate	26.002	2122.0	2018.0	20.6	0.98	$1.90*10^6$
ß-Eudesmol	27.632	2245.6	2266.0	82.8	0.72	$1.39*10^6$
3,7,11-trimethyl-6,10-Dodecadien-1-yn-3-ol	29.227	2372.0	-	46.4	0.91	$1.76*10^6$

Table S2. Gingerol-related compounds in ginger extract identified by HPLC-MS analysis.

Tentative Identification	retention time<br>min	(+) ESI-MS<br>m/z	Adduct Ion	In-source CID main fragments<br>m/z
[4]-Gingerol	6.1	249	[M+H–H2O]+	177
[6]-Gingerol	8.49	277<br>312<br>317	[M+H–H2O]+<br>[M+NH4]+<br>[M+Na]+	177
Methyl [6]-gingerol	9.9	291<br>326<br>331	[M+H–H2O]+<br>[M+NH4]+<br>[M+Na]+	191
Diacetoxy-[4]-gingerdiol	10.48	370	[M+NH4]+	293, 233
[8]-Gingerol	11.35	305<br>340<br>345	[M+H–H2O]+<br>[M+NH4]+<br>[M+Na]+	177
Acetoxy-[6]-gingerol	11.41	354	[M+NH4]+	305, 177
[6]-Shogaol	12	277	[M+H]+	
Diacetoxy-[6]-gingerdiol	13.14	398<br>403	[M+NH4]+<br>[M+Na]+	321,261,177
1-Dehydro-[6]-gingerdione	13.62	291	[M+H]+	177
[10]-Gingerol	14.25	333<br>368<br>373	[M+H–H2O]+<br>[M+NH4]+<br>[M+Na]+	333, 177
Methyl diacetoxy-[6]-gingerdiol	14.74	412<br>417	[M+NH4]+<br>[M+Na]+	335, 275, 177
[8]-Shogaol	14.96	305	[M+H]+	
Acetoxy-[10]-gingerol	16.96	410<br>415	[M+NH4]+<br>[M+Na]+	333
[12]-Gingerol	17.15	361<br>401	[M+H–H2O]+<br>[M+Na]+	177

Table S3. List of *Homo sapiens* primers used in RT-qPCR experiments.

gene	forward primer (5′-3′)	reverse primer (5′-3′)	accession number
HPRT1	GACCCCACGAAGTGTTGGAT	ACTGGCGATGTCAATAGGACTC	NM_000194
GAPDH	TGGTATCGTGGAAGGACTCA	CAGTGAGCTTCCCGTTCAG	NM_002046
TRPV6	ACACTGCTCATGCTCAAC	GATCTTGCCTGTCTTCCAC	NM_018646.6
CLDN2	TTCCCTGTTCTCCCTGATAG	CCCTGGTTCTTCACACATAC	NM_001171095.2
HMGCR	CAGAGCAAGCACATTAGCA	CAGCCAAAGCAGCACATA	NM_000859.3
SQLE	CACTTACTGGTGGAGGAATG	AGAGCCTGAGCAAGGATA	NM_003129.4
VDR	CTTTGGGTCTGAAGTGTCTG	TCACAGGTCATAGCATTGAAG	NM_001364085.2
CYP2R1	CCATTCCTAAAGGCACAACAG	AGCCAAGTGTTCTCCAAGA	NM_024514.5
CYP27A1	GCTGTTCGTTCAAGGCTAT	TCCATGTCGTTCCGTACT	NM_000784.4
CYP27B1	TGTCCAACACGCTCTCTT	GGTACAGTCTTAGCACTTCCT	NM_000785.4
CYP24A1	GAAAGAGGCCACGTTGAA	GACCATCATCCTCCCAAAC	NM_000782.5

3 Supplementary figures

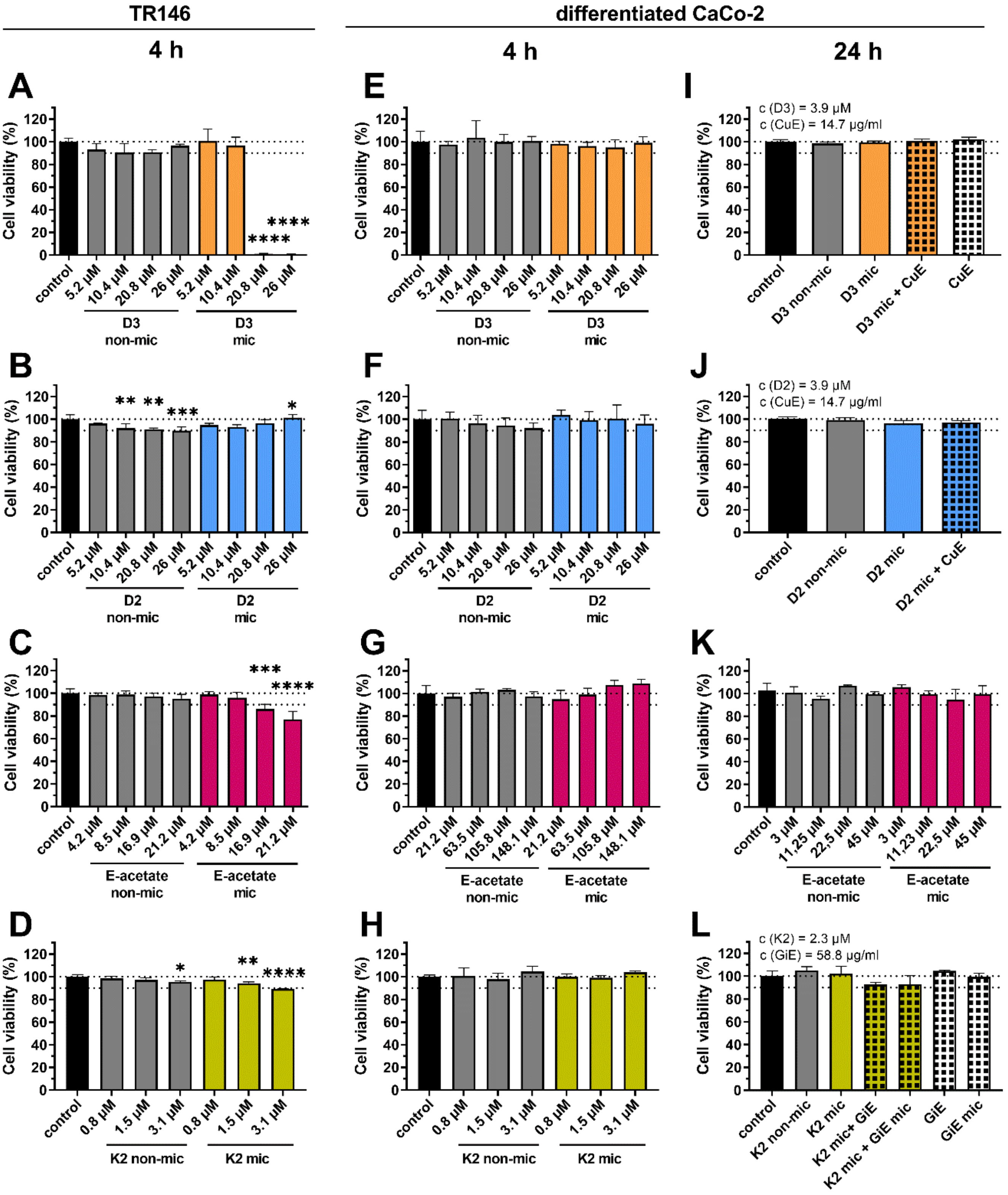

Figure S1. Cytotoxicity testing of vitamins and extracts used in uptake and bioefficacy studies. TR146 or differentiated CaCo-2 cells were treated with vitamins or extracts or a combination of both for 4 h (A-H) or 24 h (I-L) in the indicated concentrations, subsequently cell viability was determined via the resazurin assay. Data are from one experiment performed in triplicates.

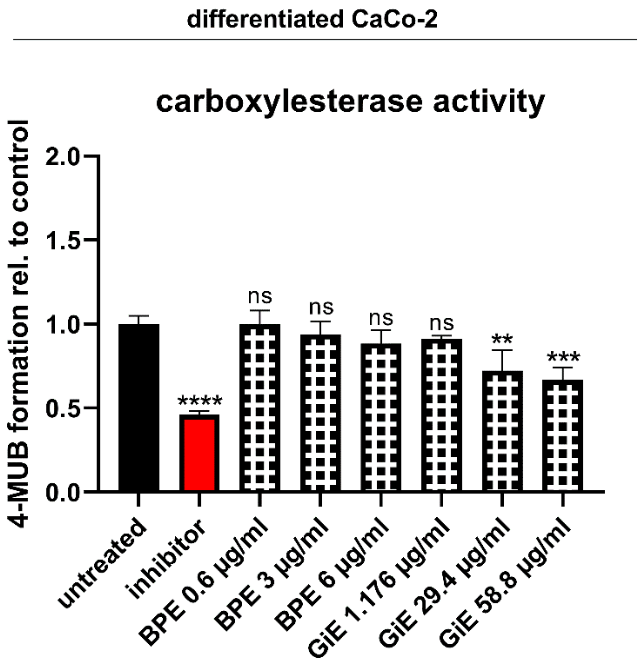

Figure S2. Effects of black pepper extract and ginger extract on carboxylesterase activity. Hydrolysis of 4-MUBA to 4-MUB by carboxylesterases (CES) from lysate of differentiated CaCo-2 cells was measured fluorometrically. Treatment was performed with the CES-inhibitor BNPP (0.5 mM), BPE or GiE at the indicated concentrations. Data shown are from one experiment performed in triplicates.

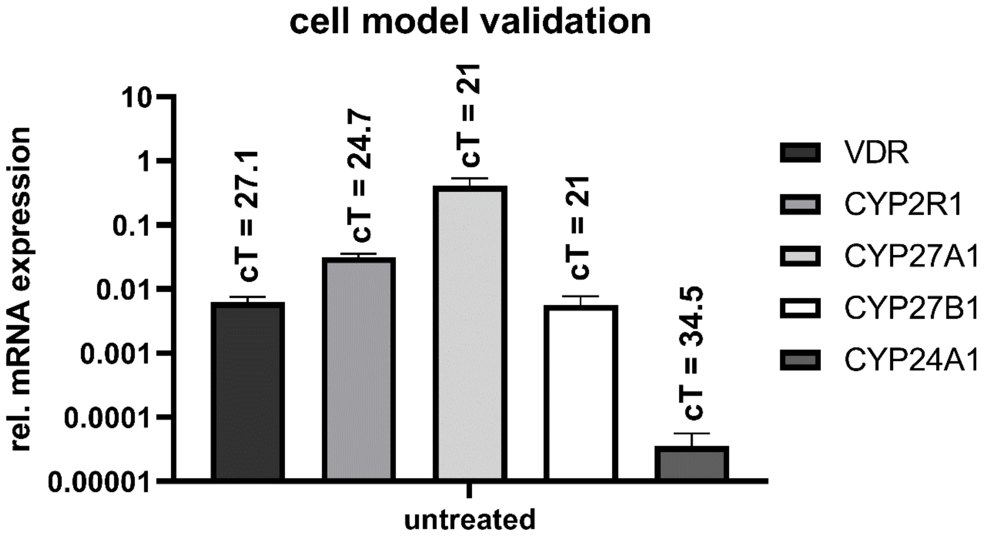

Figure S3. Validation of Caco-2 cells as model for vitamin D3/D2 effects. Differentiated Caco-2 cells were treated with control medium (MEM Eagle containing 5 mg/ml BSA) for 24 h ("untreated"). Subsequently, RNA was extracted and analyzed for expression of the VDR and vitamin D (de)activation enzymes (CYP2R1, CYP27A1, CYP27B1, CYP24A1). The target gene's expression levels were normalized to the mean of two reference genes (*GAPDH*, *HPRT1*) ($2^{-\Delta C_T}$). Mean cT values measured for each target gene are shown above each bar. Data are from two independent experiments performed in duplicates.

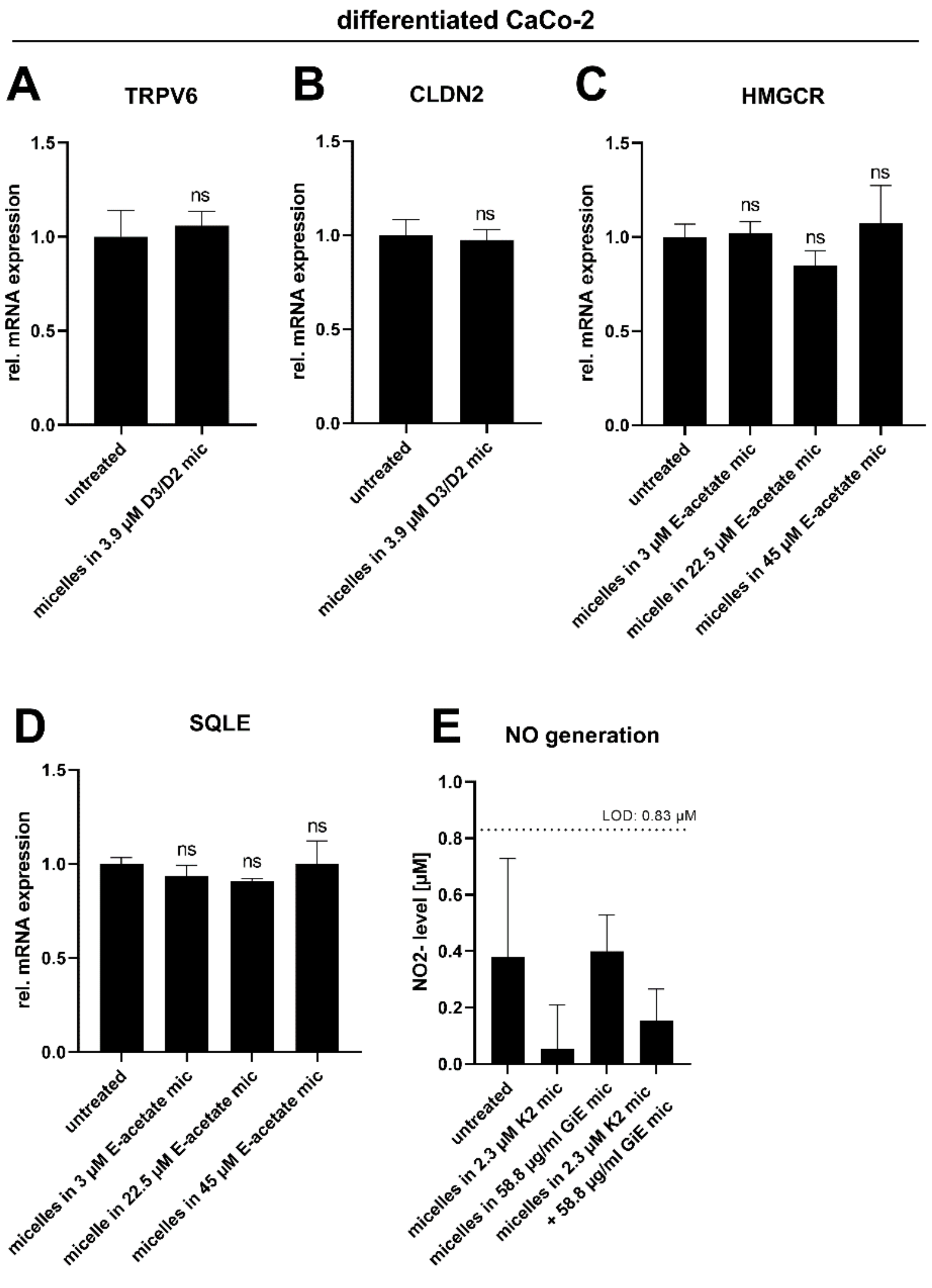

Figure S4. Effects of micelles alone on biological targets. Differentiated CaCo-2 cells were treated with micelles alone in the same amount as added in functional studies. Data are from two independent experiments performed in duplicates (A-D) or triplicates (E).

4 References

1. Blank-Landeshammer, B.; Klanert, G.; Mitter, L.; Turisser, S.; Nusser, N.; König, A.; Iken, M.; Weghuber, J. Improved Bioavailability and Bioaccessibility of Lutein and Isoflavones in Cultured Cells In Vitro through Interaction with Ginger, Curcuma and Black Pepper Extracts. *Antioxidants (Basel)* **2022**, *11*, doi:10.3390/antiox11101917.
2. MassBank consortium and its contributors. *MassBank/MassBank-data: Release version 2024.11*, 2024.
3. Jiang, H.; Sólyom, A.M.; Timmermann, B.N.; Gang, D.R. Characterization of gingerol-related compounds in ginger rhizome (Zingiber officinale Rosc.) by high-performance liquid chromatography/electrospray ionization mass spectrometry. *Rapid Commun. Mass Spectrom.* **2005**, *19*, 2957–2964, doi:10.1002/rcm.2140.
4. Lamego, J.; Ferreira, P.; Alves, M.; Matias, A.; Simplício, A.L. Comparison of in vitro methods for carboxylesterase activity determination in immortalized cells representative of the intestine, liver and kidney. *Mol. Cell. Probes* **2015**, *29*, 215–222, doi:10.1016/j.mcp.2015.05.002.
